# Bilateral calf chronic compartment syndrome in an elderly male: a case report

**DOI:** 10.4076/1757-1626-2-8346

**Published:** 2009-06-24

**Authors:** Keith Siau, Killian P O‘Rourke, Arun Khanna, Cathy J Laversuch

**Affiliations:** Department of Rheumatology, Musgrove Park HospitalTaunton, TA1 5DAUK

## Abstract

Leg pain is a common presentation to the outpatient department. Bilateral calf chronic compartment syndrome is a rare cause of bilateral calf pain. Although this condition has been well documented in young athletes, it has rarely been reported in the elderly. We present the case of a 68-year-old male bodybuilder with bilateral calf chronic compartment syndrome, describe the presentation and evaluation of the condition, and provide a review of the literature herewith.

## Introduction

A well-toned 68-year-old British Caucasian male was referred to the rheumatology department with a 4 month history of progressive, bilateral anterior and posterior calf tightness and cramping which was exacerbated by exertion and had recently been associated with mild symmetrical sensory loss in the feet. He was an ex-smoker who had previously been diagnosed with hypertension and widespread osteoarthritis affecting bilateral wrists, shoulders, hips, patellofemoral joints and lumbosacral spine - all of which were attributed to his history of weight-lifting. There were no symptoms suggestive of myopathy or sciatica. He denied illicit drug use.

**Figure 1.**
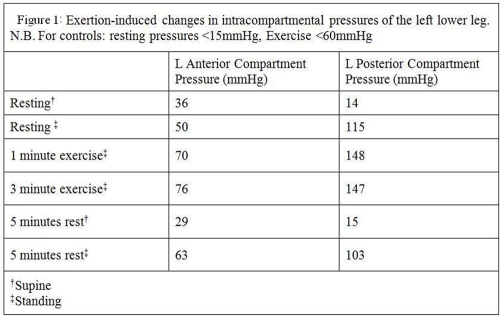
Exertion induced changes in intracompartmental pressures of the lower leg.

Examination revealed bilateral calf tenderness and mild sensory loss at the second and third toe web spaces. Lower limb power was normal with strong pedal pulses. Baseline blood tests were in keeping with his appreciable muscle bulk, revealing an elevated creatine kinase level of 561, urea of 6.8 with creatinine of 125. Serum electrolytes, D-Dimer, erythrocyte sedimentation rate, C-reactive protein and autoimmune profile were normal. Lower limb radiographs, magnetic resonance imaging of the lumbar spine (to exclude spinal stenosis), and electromyography were unremarkable. In view of the patient’s physique, he was referred for compartment pressure testing (Figure 1), which confirmed the diagnosis of bilateral calf chronic compartment syndrome (CCS).

Although an orthopaedic referral was made for consideration of bilateral fasciotomy, the patient opted for conservative treatment with simple analgesia and avoidance of weightlifting. At 6 month follow-up, his symptoms had abated without requiring surgical intervention.

## Discussion

**Figure 2.**
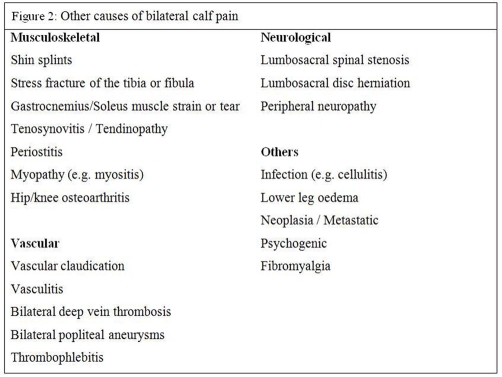
Causes of bilateral calf pain.

Leg pain is a common presentation to the outpatient department. Causes of bilateral calf pain are shown in Figure 2.

First described by Mavor in 1956 [1], CCS is an uncommon cause of bilateral calf pain characterised by exertion-induced increases in pressure within a confined anatomical space, thereby resulting in reduced perfusion to structures within the compartment. The pain of CCS is generally described as an ache or tightness over the affected compartment(s), is often proportional to the level of exertion, and progresses in severity over weeks or months [2]. Many report numbness or paraesthesiae radiating to the lower extremities [3]. The affected compartment is involved bilaterally in 82% of cases, with the smaller anterior and lateral compartments of the calf frequently being implicated [1]. CCS is well described in young athletes such as endurance runners but rarely in bodybuilders, suggesting that the pathophysiology may be more complex than large muscle bulk *per se*, and has been postulated to require a combination of reduced compartmental compliance, e.g. due to thickened fascia, [3] transient muscle hypertrophy (up to 20% during exercise), and oedema generated from repetitive hard surface contact [4]. Diagnosis can usually be made on clinical grounds, although intracompartmental pressure measurement is the gold standard [3]. Symptoms may be effectively relieved by cessation of the offending activity, but fasciotomy or fasciectomy remain the only definitive treatment [2]. There is no evidence to support the efficacy of physical therapy, orthoses or diuretics in the long term [4].

We performed a literature search on PubMed, EMBASE, and Google using the keywords: compartment syndrome AND legs/lower limbs/calf, and found no previous case reports describing non-traumatic, *bilateral* calf CCS in the over-65s. CCS in the elderly has been reported once previously by Lutz *et al* in 1988, [5] who detailed *unilateral* involvement of the anterolateral calf in a 69 year old retired coal miner who suffered from progressive exertion-induced calf pain for 4 years prior to diagnosis, and promptly became symptom-free after fasciotomy. From the largest series of CCS patients involving 100 young patients, Detmer *et al* reported that the average duration from presentation to diagnosis was 22 months, with each patient consulting an average of 2.4 physicians before the diagnosis was made, [2] illustrating the underdiagnosis and poor awareness of the condition. Physicians should thus consider CCS as a cause of bilateral calf pain as the condition is readily amenable to treatment, and early recognition is important to minimise morbidity.

## Abbreviation

CCS: Chronic compartment syndrome
